# Dynamic Change in Mean Platelet Volume and Delayed Cerebral Ischemia After Aneurysmal Subarachnoid Hemorrhage

**DOI:** 10.3389/fneur.2020.571735

**Published:** 2020-11-30

**Authors:** Liuwei Chen, Quanbin Zhang

**Affiliations:** Department of Neurosurgery, Shanghai Tenth People's Hospital, Tongji University School of Medicine, Shanghai, China

**Keywords:** subarachnoid hemorrhage, mean platelet volume, delayed cerebral ischemia, hypercoagulability, hemorrhagic stroke

## Abstract

**Background:** The mean platelet volume (MPV) has been shown to predict short-term outcomes in patients who have experienced aneurysmal subarachnoid hemorrhage (aSAH). The purpose of this study was to explore the temporal variation of MPV in patients with aSAH and its relationship to the development of delayed cerebral ischemia (DCI).

**Methods:** Data from 197 consecutive aSAH patients who were treated at our institution between January 2017 and December 2019 were collected and analyzed. Blood samples to assess MPV were obtained at 1–3, 3–5, 5–7, and 7–9 d after the initial hemorrhage. Univariate and multivariate analyses were performed to investigate whether MPV was an independent predictor of DCI and the receiver operating characteristic (ROC) curve and area under the curve (AUC) were determined.

**Results:** The MPV values in patients with DCI were significantly higher compared to those without DCI at 1–3, 3–5, 5–7, and 7–9 d after hemorrhage (*P* < 0.001). The trend for MPV in patients with DCI was increased at first and then decreased. The transition from increases to decreases occurred at 3–5 d after hemorrhage. The optimal cutoff value for MPV to accurately predict DCI was 10.35 fL at 3–5 d after aSAH in our cohort. Furthermore, the MPV observed at 3–5 d was an independent risk factor for DCI [odds ratio (OR) = 4.508, 95% confidence interval (CI): 2.665–7.626, *P* < 0.001].

**Conclusions:** MPV is a dynamic variable that occurs during aSAH, and a high MPV at 3–5 days after hemorrhage is associated with the development of DCI.

## Introduction

Aneurysmal subarachnoid hemorrhage (aSAH) is a critical hemorrhagic stroke with a high risk of morbidity, mortality, and associated financial costs (1, 2). The mechanism of brain injury following aSAH remains multifactorial. There is evidence that thrombosis and inflammation may be elevated following aSAH (3, 4).

Delayed cerebral ischemia (DCI) is a major cause of poor functional outcomes in patients who survive following an initial hemorrhage (5, 6). DCI has been associated with a worse prognosis, a more severe clinical course, and increased mortality in patients with aSAH (7). The pathogenesis of DCI involves micro-thrombosis, neurovascular uncoupling, perfusion mismatch, spreading depolarizations, and inflammatory responses, all of which culminate in infarction (8). Identifying patients who are at high risk of DCI will assist with the management of patients with aSAH.

Mean platelet volume (MPV) reflects the average size of platelets, which is associated with the function and activation of platelets (9). Previous studies have demonstrated the clinical significance of MPV in many thrombotic diseases, such as acute ischemic stroke (10), acute myocardial infarction (11), and deep vein thrombosis (12). Our initial work also showed that an elevated MPV was associated with poor short-term outcomes in aSAH patients (13). However, there is few published research concerning the dynamic change in MPV during aSAH and its relationship with DCI (14). The purpose of this study was to explore the temporal variation of MPV in patients with aSAH and the relationship of MPV with the development of DCI.

## Methods

### Patients

Data from consecutive aSAH patients who were treated at our institution between January 2017 and December 2019 were collected and analyzed. The inclusion criteria for enrollment were as follows. (1) aSAH was confirmed by digital subtraction angiography or CT angiography. (2) Patients had received surgical clipping or endovascular coiling within 48 h after admission. (3) The time between symptom onset and admission was <24 h.

The study exclusion criteria were as follows. (1) The patients presented with other cerebral vascular diseases, such as Moyamoya disease and arteriovenous malformation. (2) The patients experienced prior systemic diseases, such as hematological disease, immunological disease, recent infectious disease, or severe hepatic and renal dysfunction. (3) The patient died within 3 days after admission or declined medical intervention; (4) The patients had missing data. The study protocol was approved by the ethics committee.

### Clinical Protocol and Laboratory Tests

Demographic and clinical data were collected from patients, including sex, age, medical history, clinical status on admission (Hunt-Hess grade on admission), radiological characteristics [modified Fisher grade on admission, acute hydrocephalus, intracerebral hemorrhage (ICH)], treatment method (coiling or clipping), aneurysm size, aneurysm location, and development of DCI. DCI was defined according to the AHA/ASA definition (15) as the occurrence of focal neurological impairment or a decrease in at least two points on the Glasgow Coma Scale, which was not apparent immediately after aneurysm occlusion, and could not be attributed to other causes by means of clinical assessment, imaging of the brain, and appropriate laboratory studies. The management of aSAH patients was carried out according to the aSAH management guideline (16, 17). For interpretation purposes, some parameters were dichotomized as follows, Hunt-Hess grade as “grade 1–3” and “grade 4–5,” modified Fisher grade as “grade 0–2” and “grade 3–4,” treatment method as “coiling” and “clipping,” aneurysm size as “ <5 mm,” “5–10 mm,” and “>10 mm,” and aneurysm location as “internal cerebral artery,” “anterior cerebral artery,” “middle cerebral artery,” and “vertebrobasilar artery.”

Blood samples were obtained from all patients at 1–3, 3–5, 5–7, and 7–9 d after the initial hemorrhage. Samples were stored at room temperature in anticoagulant tubes and were analyzed within 1 h after venipuncture. MPV and platelet (PLT) counts were determined using an autoanalyzer (Sysmex Company, XE-2100, Japan). The MPV:PLT ratio was calculated as MPV/PLT ^*^100.

### Statistical Analysis

Statistical analyses were performed using SPSS version 21.0 (SPSS, Chicago, IL, USA). Continuous variables were presented as medians (interquartile range, IQR), and categorical variables were expressed as numbers (frequencies). In the univariate analysis, continuous variables were compared using the Mann–Whitney *U*-test and categorical variables were compared using the χ^2^-test or Fisher exact test. Variables with a *p* < 0.10 in the univariate analysis were entered into a multivariate regression model. The relationship of MPV to DCI was investigated using the multivariate regression model. The receiver operating characteristic (ROC) curve analysis was performed to assess the overall discrimination ability of MPV to predict DCI and to establish an optimal cutoff value using the Youden index. The DeLong method was used to compare the differences in discriminative ability. A two-tailed value of *P* < 0.05 was considered to be significant.

## Results

**Figure 1:**
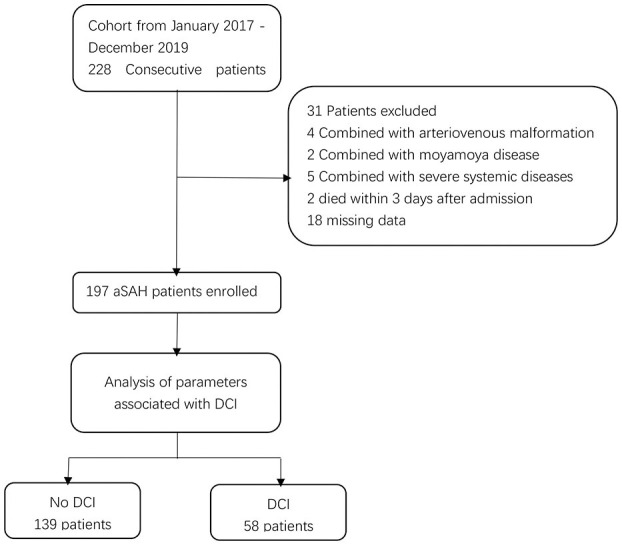
Flowchart illustrating the patient cohort included in this study. aSAH indicates aneurysmal subarachnoid hemorrhage; DCI indicates delayed cerebral ischemia.

A total of 197 patients [114 females [57.9%]] were included in this study (Figure 1). The median age at the time of admission was 60 years (IQR: 51–67). A total of 155 (78.7%) patients received endovascular coiling, and 42 (21.3%) received surgical clipping. Overall, 58 (29.4%) patients developed DCI. Patients were divided into two groups according to the occurrence of DCI. The baseline characteristics are presented in Table 1. Univariate analysis showed that patients with DCI had a higher Hunt-Hess grade, higher modified Fisher grade, a higher incidence of clipping was used, and a higher incidence of acute hydrocephalus and ICH compared to patients without DCI.

**Table 1:** Baseline characteristics of patients according to the development of delayed cerebral ischemia.

**Variable**	**Total**	**DCI**		***P*-value**
	**(*****n*** **=** **197)**	**Yes (*****n*** **=** **58)**	**No (*****n*** **=** **139)**	
Age (years)	60 (51–67)	61 (53–66)	59 (48–67)	0.295
Sex (female)	114 (57.9%)	30 (51.7%)	84 (60.4%)	0.259
Hypertension	113 (57.4%)	32 (55.2%)	81 (58.3%)	0.688
Diabetes	30 (15.2%)	11 (19.0%)	19 (13.7%)	0.346
Hunt-Hess grade				
Grade 1, 2, 3	148 (75.1%)	28 (48.3%)	120 (86.3%)	<0.001
Grade 4, 5	49 (24.9%)	30 (51.7%)	19 (13.7%)	
Modified Fisher grade				<0.001
Grade 0, 1, 2	62 (31.5%)	5 (8.6%)	57 (41.0%)	
Grade 3, 4	135 (68.5%)	53 (91.4%)	82 (59.0%)	
Location				0.403
ACA	72 (36.5%)	22 (37.9%)	50 (36.0%)	
ICA	70 (35.5%)	20 (34.5%)	50 (36.0%)	
MCA	28 (14.2%)	11 (19.0%)	17 (12.2%)	
VBA	27 (13.7%)	5 (8.6%)	22 (15.8%)	
Size (mm)				0.530
<5	112 (56.9%)	30 (51.7%)	82 (59.0%)	
5–10	67 (34.0%)	21 (36.2%)	46 (33.1%)	
>10	18 (9.1%)	7 (12.1%)	11 (7.9%)	
Treatment				0.032
Coiling	155 (78.7%)	40 (69.0%)	115 (82.7%)	
Clipping	42 (21.3%)	18 (31.0%)	24 (17.3%)	
Acute hydrocephalus	25 (12.7%)	17 (29.3%)	8 (5.8%)	<0.001
ICH	42 (21.3%)	23 (39.7%)	19 (13.7%)	<0.001

*Data are n (%), or median (interquartile)*.

*DCI, delayed cerebral ischemia; ACA, anterior cerebral artery; ICA, internal carotid artery; MCA, middle cerebral artery; VBA, vertebrobasilar artery; ICH, intracerebral hemorrhage*.

**Figure 2:**
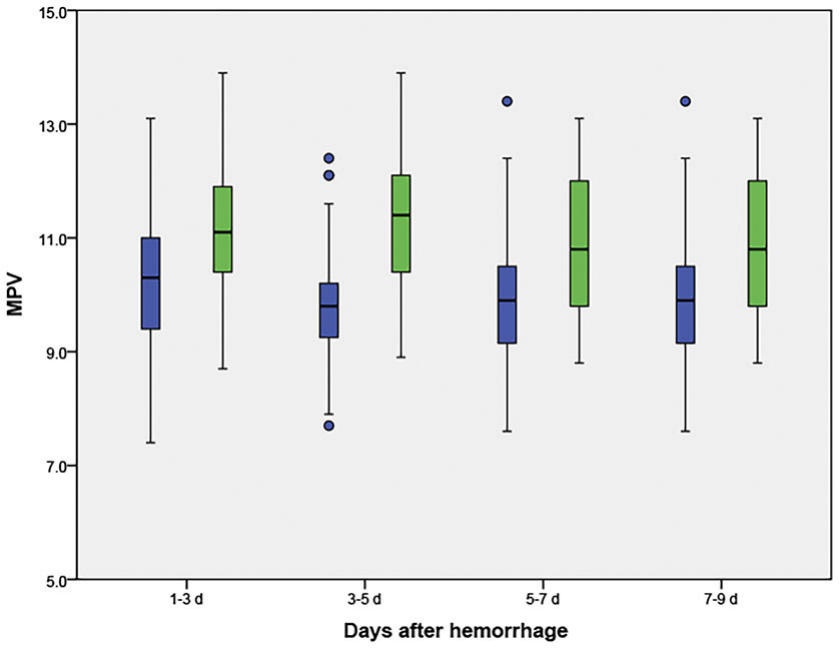
Temporal profile for the mean platelet volume (MPV) in aneurysmal subarachnoid hemorrhage patients, according to the occurrence of delayed cerebral ischemia (DCI). Blue boxes indicate patients without DCI; green boxes indicate patients with DCI.

**Figure 3:**
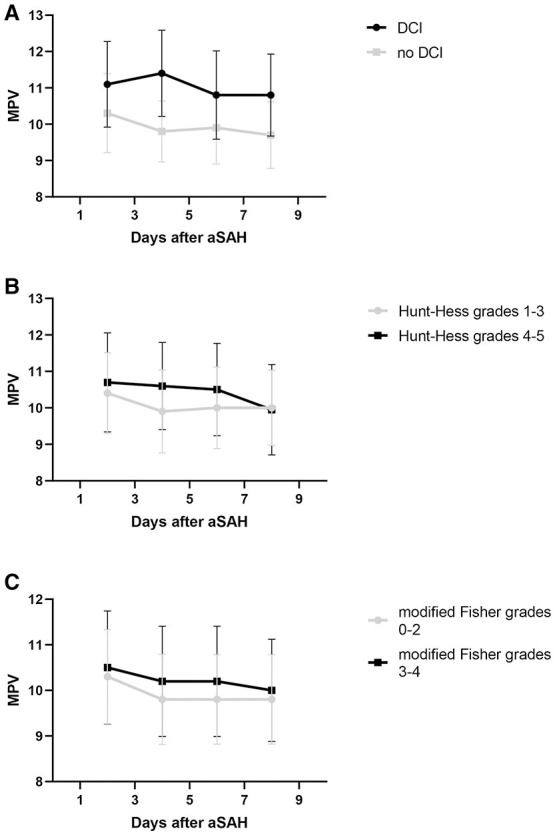
Trend of mean platelet volume (MPV) after aneurysmal subarachnoid hemorrhage (aSAH). Whisker bars represent the standard error of the mean. **(A)** Graph showing the temporal profile of MPV in patients delineated by the occurrence of delayed cerebral ischemia (DCI). **(B)** Graph showing the temporal profile of MPV in patients delineated by their Hunt-Hess grade. **(C)** Graph showing the temporal profile of MPV in patients delineated by their modified Fisher grade.

The MPV was obtained at four different time points: 1–3, 3–5, 5–7, and 7–9 d after hemorrhage. The temporal profiles of the MPV according to the occurrence of DCI are shown in Figure 2. The MPV values in patients with DCI were significantly higher compared to those without DCI at 1–3, 3–5, 5–7, and 7–9 d after hemorrhage (*P* < 0.001, Table 2). The trend for MPV in patients with DCI increased at first and then decreased, with the transition occurring at 3–5 d after hemorrhage. A decreasing trend in the MPV was observed in patients without DCI (Figure 3A). A decreasing trend in the MPV also was observed in patients segregated according to their Hunt-Hess grade and modified Fisher grade (Figures 3B,C).

**Table 2:** Temporal profile of plasma MPV, PLT, and MPV:PLT ratio in aSAH patients regarding the development of DCI.

	**DCI**	**No DCI**	***P*-value**
1–3 d			
MPV	11.1 (10.4–11.9)	10.3 (9.4–11.0)	<0.001
PLT	175 (144–232)	195 (163–232)	0.051
MPV:PLT ratio	6.40 (4.78–8.67)	5.24 (4.33–6.36)	0.003
3–5 d			
MPV	11.4 (10.4–12.1)	9.8 (9.2–10.2)	<0.001
PLT	161 (118–191)	177 (147–220)	0.003
MPV:PLT ratio	6.92 (5.49–9.68)	5.48 (4.35–6.67)	<0.001
5–7 d			
MPV	10.8 (9.8–11.9)	9.9 (9.1–10.5)	<0.001
PLT	157 (117–208)	197 (163–243)	<0.001
MPV:PLT ratio	6.43 (4.88–10.01)	5.05 (4.06–6.12)	<0.001
7–9 d			
MPV	10.8 (9.8–11.6)	9.7 (9.1–10.5)	<0.001
PLT	196 (138–240)	217 (180–275)	0.013
MPV:PLT ratio	5.1 (4.42–8.35)	4.47 (3.57–5.46)	0.001

*Data are median (interquartile)*.

*MPV, mean platelet volume; PLT, platelet; DCI, delayed cerebral ischemia*.

**Figure 4:**
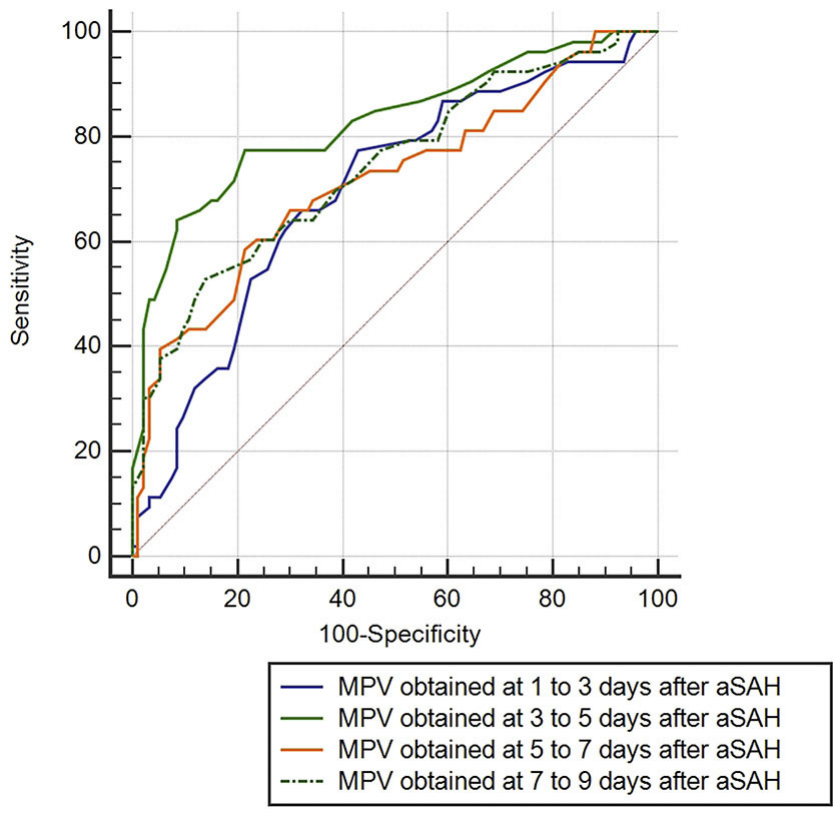
Discrimination ability of the mean platelet volume (MPV) for delayed cerebral ischemia (DCI).

**Figure 5:**
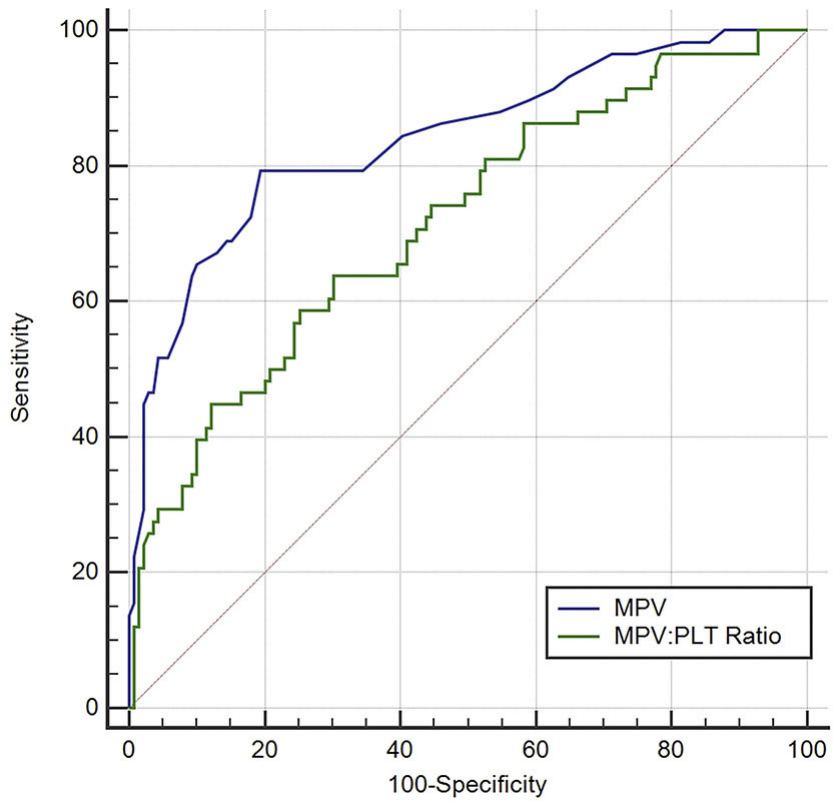
Discrimination ability of the mean platelet volume (MPV) for delayed cerebral ischemia (DCI), compared with the MPV: platelet ratio. PLT indicates platelet.

ROC curve analysis found that MPV observed 3–5 d after aSAH could predict DCI with an area under the curve (AUC) of 0.826 [95% confidence interval (CI): 0.754–0.884, *P* < 0.001, Figure 4]. The AUC for MPV obtained at 3–5 d was larger than the MPV observed at other time points (Table 3). The optimal cut-off value for MPV to predict DCI was 10.35 fL at 3–5 d after aSAH, with a sensitivity of 79.3% and a specificity of 80.6%. Meanwhile, the AUC for MPV at 3–5 d was larger than that for the MPV:PLT ratio at 3–5 d [MPV at 3–5 d [AUC: 0.826, 95% CI: 0.754–0.884] vs. MPV:PLT ratio at 3–5 d [AUC: 0.716, 95% CI: 0.636–0.793], *P* < 0.001, Figure 5].

**Table 3:** Diagnostic values of MPV for DCI.

	**AUC (95% CI)**	***P***	**Cutoff value**	**Sensitivity (%)**	**Specificity (%)**
1–3 d MPV	0.696 (0.615–0.770)	<0.001	10.35	77.6%	56.8%
3–5 d MPV	0.826 (0.754–0.884)	Reference	10.35	79.3%	80.6%
5–7 d MPV	0.717 (0.636–0.788)	0.001	10.65	57.9%	81.3%
7–9 d MPV	0.740 (0.661–0.809)	0.010	10.75	52.8%	85.4%

*MPV, mean platelet volume; DCI, delayed cerebral ischemia; AUC, area under the curve; CI, confidence interval*.

Multivariate logistic regression analysis indicated that MPV at 3–5 d [odds ratio (OR) = 4.508, 95% CI: 2.665–7.626, *P* < 0.001] was an independent risk factor for DCI, with adjustment for the Hunt-Hess grade, modified Fisher grade, treatment methods, acute hydrocephalus, ICH, PLT, and MPV:PLT ratio (Table 4).

**Table 4:** Multivariate logistic analysis of predictors for delayed cerebral ischemia.

**Predictors**	**OR (95% CI)**	***P*-value**
Hunt-Hess grade 4 and 5	2.406 (0.783–7.388)	0.125
Modified Fisher grade 3 and 4	3.004 (0.839–10.758)	0.091
Clipping	1.332 (0.414–4.279)	0.630
Acute hydrocephalus	3.197 (0.882–11.589)	0.077
ICH	2.430 (0.765–7.724)	0.132
3–5 d MPV	4.508 (2.665–7.626)	<0.001
3–5 d PLT	1.004 (0.990–1.018)	0.582
3–5 d MPV:PLT ratio	0.987 (0.780–1.249)	0.915

*ICH, intracerebral hemorrhage; MPV, mean platelet volume; PLT, platelet; OR, odds ratio; CI, confidence interval*.

## Discussion

Our study demonstrated a correlation between MPV and DCI in aSAH patients. Specifically, aSAH patients who developed DCI had higher MPV values over time compared to patients without DCI. Second, we observed a rise and then decrease in MPV in patients with DCI with the transition occurring at 3–5 d after hemorrhage. This trend was not observed in patients without DCI. Third, multivariate regression analysis revealed that increased MPV was an independent risk factor for DCI. The best discriminating MPV value for predicting DCI was 10.35 or more at 3–5 d after hemorrhage. Consequently, a high MPV at 3–5 d after hemorrhage was associated with the development of DCI in patients with aSAH.

MPV reflects the average size of platelets and is associated with the function and activation of platelets (9). Previous studies have shown the clinical significance of MPV in many thrombotic diseases. An increased MPV at admission is associated with poor functional outcomes in patients with acute ischemic stroke (10). The rise of MPV during hospitalization also is associated with a poor long-term prognosis in patients with acute myocardial infarction (11). In patients with deep vein thrombosis, an elevated MPV is an independent risk factor of pulmonary embolism (12). Our previous study revealed that MPV has prognostic value in aSAH. An elevated MPV is associated with poor short-term outcomes in aSAH patients (13). In that study, we did not investigate the temporal variation in MPV. We explore the relationship between MPV and DCI, and describe the temporal variation in MPV during aSAH in this study.

It is well-established in the literature that patients are in a hypercoagulable state in the early phase after aSAH. aSAH patients show an increase in clot strength after symptom onset, which is driven by increased platelet activation (18, 19). Clot strength remains increased in the early period after aSAH. The level of coated-platelets also is higher in aSAH patients in the early period compared to normal controls (20). A previous study showed that the level of fibrinogen and D-dimer increased after aSAH, and both were associated with delayed ischemic neurological deficits (21). Similarly, a trend toward increasing MPV in patients with DCI was observed in our study, suggesting that larger and perhaps more reactive platelets were circulating in those patients in the early phase after aSAH. However, this trend toward increased MPV did not exist in patients without DCI or when segregated by the Hunt-Hess grade or modified Fisher grade. Our results add to the growing evidence that a hypercoagulable state might contribute to the development of DCI.

We postulate a potential reason why the enhanced MPV is associated with DCI. First, an elevated MPV reflects a higher thrombotic potential following aSAH. An elevated MPV represents a larger platelet size, and the presence of large platelets indicates increased platelet activation. The larger platelets also have more dense granules, release more thromboxane and β-thromboglobulin, and express more procoagulant surface proteins, such as P-selectin and glycoprotein IIIa (9, 22, 23). An increased release of thromboxane and expression of P-selectin leads to the formation of micro-thromboses and arteriolar constriction following aSAH, which reduces cerebral blood flow and causes DCI (3, 24). Second, an elevated MPV reflects a severe inflammatory response following aSAH. The production of larger platelets is mediated by several cytokines, such as interleukin-3 (IL-3) and interleukin-6 (IL-6) (25). These cytokines can regulate the megakaryocyte ploidy and increase the number of megakaryocytes, which leads to the production of larger platelets. The level of IL-6 is elevated early after aSAH occurs, and it remains high during aSAH (26). A delayed increase in IL-6 is observed in aSAH patients with cerebral infarction (26). It has also been proven that elevated IL-6 is associated with the development of vasospasms after aSAH (27). The increasing concentration of IL-6 after aSAH may lead to the elevation of MPV. Taken together, the elevation of MPV indicates the production of larger and more active platelets, and it indicates the presence of a hypercoagulable state and a severe inflammatory response after hemorrhage. Meanwhile, an increased platelet size also contributes to the formation of thrombi. The associations mentioned above may explain why an elevated MPV is associated with the development of DCI. However, further experiments are needed to validate this hypothesis.

Recently, Ray et al. reported a rise and fall of the MPV:PLT ratio in patients with aSAH. They found that the MPV:PLT ratio was independently associated with the development of DCI and functional outcomes (14, 28). In our study, a similar trend of the MPV:PLT ratio was observed (see Table 2). The trend toward increasing MPV also was more pronounced in patients with DCI, which was consistent with their results. However, the AUC for the MPV at 3–5 d was larger than that for the MPV:PLT ratio at 3–5 d. In addition, the logistic regression analysis indicated that the MPV:PLT ratio was not an independent predictor of DCI. One possible reason for the difference is that the platelet count can be influenced by many iatrogenic factors, such as platelet transfusion, craniotomy, and drugs. Also, many factors influence the accuracy of MPV measurements, such as the time interval for measurement, storage temperature, and use of anticoagulants (29). In our study, some actions were taken to ensure the accuracy of MPV measurement. For example, the blood samples are stored at room temperature using anticoagulant tubes, and all the samples are analyzed within 1 h after venipuncture.

The use of transcranial doppler ultrasonography, electroencephalography, and CT perfusion imaging is recommended to monitor DCI (17). However, compared with these monitoring methods, MPV measurement is more convenient and widely available. We postulate that an elevated MPV after aSAH may represent a hypercoagulable state and a severe inflammatory response. The MPV can be used in clinical settings to estimate a patient's susceptibility to DCI and help clinicians recognize patients who are at high risk. The safety and efficacy of antiplatelet therapy in aSAH have been proven (30–32). Antiplatelet therapy can improve the functional outcome in patients and reduce their risk of DCI. When patients undergo an elevation in MPV during aSAH, it may be appropriate to start antiplatelet therapy, because these patients are likely to be in a hypercoagulable state and at high risk for DCI.

Our study had several limitations. First, it is a retrospective observational study from a single center, making it prone to bias and challenging to estimate generalizability. The time point for MPV measurement is relatively broad. Second, we did not collect data on pre-aSAH drug use, such as lipid-lowering drugs and antiplatelet drugs, which may influence the MPV value. Third, the definition of DCI, especially as applied to patients who were sedated or comatose, may have excluded some patients who developed DCI. Fourth, we didn't explore the possible mechanism of MPV in DCI. A multi-center, prospective study is needed to verify our findings in the future.

## Conclusion

In conclusion, the present study suggests that MPV is a dynamic variable that occurs during aSAH, and a high MPV at 3–5 days after hemorrhage is associated with the occurrence of DCI. These results may help guide clinicians to manage aSAH patients.

## Data Availability Statement

The raw data supporting the conclusions of this article will be made available by the authors, without undue reservation.

## Ethics Statement

The studies involving human participants were reviewed and approved by the Ethical Committee of Shanghai Tenth People's Hospital. The patients/participants provided their written informed consent to participate in this study.

## Author Contributions

QZ: conception, design, and administrative support. LC: provision of study materials or patients, collection and assembly of data, data analysis, and interpretation. All authors: manuscript writing and final approval of manuscript.

## Conflict of Interest

The authors declare that the research was conducted in the absence of any commercial or financial relationships that could be construed as a potential conflict of interest.
